# Konzept und Umsetzung eines adaptiven digitalen Hörtrainingssystems für die Cochlea-Implantatnachsorge

**DOI:** 10.1007/s00106-023-01414-7

**Published:** 2024-01-30

**Authors:** Maika Werminghaus, Florian Gnadlinger, Jutta G. Richter, André Selmanagić, Susann Thyson, Dorothee Schatton, Thomas Klenzner

**Affiliations:** 1 Klinik für Hals-Nasen-Ohrenheilkunde, Hörzentrum, Medizinische Fakultät und Universitätsklinikum Düsseldorf, Heinrich-Heine-Universität Düsseldorf, Moorenstr. 5, 40225 Düsseldorf, Deutschland; 2 Masrayk University, Brünn, Tschechien; 3 Klinik für Rheumatologie, Medizinische Fakultät und Universitätsklinikum Düsseldorf, Heinrich-Heine-Universität Düsseldorf, Düsseldorf, Deutschland; 4 Hiller Forschungszentrum Rheumatologie, Medizinische Fakultät und Universitätsklinikum Düsseldorf, Heinrich-Heine-Universität Düsseldorf, Düsseldorf, Deutschland; 5 Forschungs- & Weiterbildungszentrum für Kultur und Informatik, Hochschule für Technik und Wirtschaft Berlin, Berlin, Deutschland

**Keywords:** Lernendes Gesundheitssystem, Game based learning, Rehabilitation, eHealth, Cochlea Implantat, Learning health system, Game based learning, Rehabilitation, Digital health, Cochlea-Implantat

## Abstract

**Hintergrund und Ziel:**

Im Rahmen eines interdisziplinären Forschungsprojekts wurde ein Prototyp eines adaptiven, digitalen Hörtrainingssystems für Cochlea-Implantat(CI)-Nutzende entwickelt. Die Autoren integrierten eine dynamische Schwierigkeitsanpassung in Abhängigkeit von der individuellen Leistung des Nutzenden unter Verwendung des „Evidence-Centered-Design(ECD)-Frameworks“.

**Methoden:**

Das ECD bietet einen konzeptionellen Gestaltungsrahmen, der sich für komplexe Beurteilungen von Kompetenzen und dynamischen Leistungen eignet. In der Findungsphase wurden zunächst die Teilbereiche des Hörens im Kontext von CI-Nutzenden definiert. In der Entwicklungsphase wurden das im ECD vorgesehene Kompetenzmodell, das Evidenzmodell sowie ein Aufgabenmodell entwickelt und implementiert. Zusätzlich wurde ein Assetpool mit Sound- und Sprachdateien angelegt, der umfassende linguistische Merkmalsbeschreibungen zur Berechnung der Itemschwierigkeiten beinhaltete.

**Ergebnisse:**

Aufgrund der beschriebenen Anforderungen wurden ein adaptiver Übungsgenerator, ein Künstlicher-Intelligenz(KI)-Service sowie weitere Komponenten implementiert. Dies umfasste die Entwicklung eines Spielumfelds und eines Dashboards für das Patientendatenmanagement. Für die Berechnung des Schwierigkeitsgrads der Übungen wurden die Itemschwierigkeiten anhand verschiedener Parameter (z. B. Klang, Worthäufigkeit und Anzahl der Wörter, grammatische Eigenschaften) in Kombination mit definierten Aufgabentypen und -leveln bestimmt.

**Schlussfolgerung:**

Die Nachsorge von CI-Patienten kann durch ein adaptives digitales Hörtrainingssystem in einem kontinuierlichen, interaktiven Prozess unter Berücksichtigung individueller Bedürfnisse gewinnbringend erweitert werden. Die Autoren sehen das ECD als einen effektiven Weg, ein benutzerbasiertes, anpassungsfähiges System aufzubauen.

## Abstract (en)

**Background and objective:**

Within the context of an interdisciplinary research project, we created a cutting-edge prototype of an adaptive digital auditory training system designed for cochlear implant (CI) users. By leveraging the evidence-centered design (ECD) framework, we integrated a dynamic difficulty adjustment feature that tailors the experience to the unique performance capabilities of each individual user.

**Methods:**

The ECD provides a conceptual design framework suitable for complex assessments of competence and dynamic performance. In the first phase, the domain of hearing was first defined in the context of CI users. In the development phase the three core models of the ECD, the competence model, the evidence model, and the task model, were developed and implemented. In addition, an asset pool of sound and language files was created, which included comprehensive linguistic feature descriptions for calculating item difficulties.

**Results:**

Based on the requirements described, an adaptive exercise generator, an AI service, and other components were implemented. This included the development of a game environment and a dashboard for patient data management. The exercises’ difficulty levels were determined based on various parameters (e.g., sound, word frequency and number of words, grammatical properties) in combination with defined task types and levels.

**Conclusion:**

An adaptive digital auditory training system can help to supervise and train CI patients in a continuous, interactive process based on their individual needs. We see the ECD as an effective way to build a user-based adaptive system.

Im Zuge der Digitalisierung steigt die Nutzung digitaler Anwendungen auch im medizinisch-therapeutischen Bereich. Ein digitales Hörtraining soll aus Patientensicht Freude bereiten und gleichzeitig effektiv sein. Im Folgenden wird ein System für ein adaptives, digitales Hörtraining dargestellt, das ein durch künstliche Intelligenz (KI-)gestütztes Verfahren nutzt. Im Fokus der Entwicklung stand ein Prototyp für ein digitales Hörtrainingssystem, der an die Hörkompetenzen der Patienten angepasste Übungsaufgaben auswählen kann.

## Digitales Hörtrainingssystem

In Deutschland ist eine an die Cochlea-Implantation(CI)-Operation angeschlossene Nachsorge in ambulanter oder stationärer Form laut der aktuellen AWMF-Leitlinie (Arbeitsgemeinschaft der Wissenschaftlichen Medizinischen Fachgesellschaften e. V.) aus dem Jahr 2022 obligatorisch [16]. In der hör- und sprachtherapeutischen Basis- und Folgetherapie werden Übungen in direkter Interaktion von Therapeut und Patient durchgeführt. Digitale Übungsangebote (z. B. in Form von Apps) werden noch selten in das Hörtraining eingebunden [24, 26]. Verordnungsfähige digitale Gesundheitsanwendungen (DiGA, Definition s. Tab. 2) zum Thema Hörtraining sind im DiGA-Verzeichnis des Bundesinstituts für Arzneimittel und Medizinprodukte (BfArM) nicht verzeichnet [3]. Mit dem technologischen Fortschritt und dem wachsenden Angebot an eHealth-Anwendungen steigt auch bei den CI-Patienten das Interesse an patientenzentrierten, digitalen Übungsmöglichkeiten zu ihrer Höroptimierung.

Die Hörerfahrungen und demografischen Eigenschaften sind ebenso heterogen wie das Outcome von CI-Patienten [6]. Bisherige Hörtrainings-Apps beinhalten jedoch noch wenig adaptive oder automatisierte Systeme, sodass eine Anpassung der Übungseinheiten an das Hörkompetenzniveau des Einzelnen bisher nicht gegeben ist [24, 26]. Die besondere Anforderung an ein effektives digitales Hörtrainingssystem liegt darin, die Hörkompetenz der Patienten zu erkennen und Übungen zu generieren, die ihrem individuellen Leistungsniveau entsprechen.

Im interdisziplinären Netzwerkprojekt ProWear:Cochlea wurde eine prototypische Applikation für mobile Endgeräte entwickelt und in einer klinischen Machbarkeitsstudie erprobt. Ziel war die Entwicklung einer neuartigen, sich adaptiv und dynamisch an die Bedürfnisse der Patienten anpassenden Hörtrainings-App. Die klinischen Daten werden in einem separaten Paper in Kürze vorgestellt. Die Trainingsapplikation wurde in Form eines Serious Games (Definition s. Tab. 2) konzipiert. So wurde eine sinnvolle Verknüpfung zwischen Hörrehabilitation und spielerischen Elementen hergestellt, um sicherzustellen, dass der Patient die Übungen in möglichst umfassendem Maße durchführt und seine Adhärenz erhöht wird. Die Förderung von Motivation und positiven Emotionen während des Spielens trägt dazu bei, dass Lerninhalte effektiver erfasst werden [15].

## Methode

Die technologische Umsetzung eines adaptiven Lernkonzepts bedeutet i. Allg., dass der zu lernende Inhalt sich an die individuellen Voraussetzungen des Lernenden anpasst [12]. Die im Projekt gewählte Adaption basierte auf der Auswahl des Schwierigkeitsgrads des jeweiligen Übungstyps gemessen an der Fähigkeit des Patienten. Die Entwicklung des Hörtrainingssystems fußte auf dem „Evidence-Centered Design“ (ECD). Das ECD stellt ein konzeptionelles Design-Framework dar, das ursprünglich für Assessments im schulischen Kontext entwickelt wurde [17]. Innerhalb des Frameworks interagieren verschiedene Komponenten zirkulär miteinander, sodass ein ständiges Anpassen an die aktuellen Fähigkeiten des Patienten gewährleistet werden konnte. Ein auf künstlicher Intelligenz (KI) beruhender Service bestimmt dabei anhand der angenommenen Kompetenzen des Nutzers die Schwierigkeit der nächsten Aufgabe. Das Serious Game präsentiert die Aufgabe in der spielerischen Umgebung, die durch den Nutzer ausgewählt wurde. Es liefert die Ergebnisse zurück an den KI-Service, wodurch die Kompetenzeinschätzung aktualisiert und eine nächste Aufgabenschwierigkeit vorschlagen wird. Die verwendeten Komponenten wurden spezifisch an die Anforderungen eines klinischen Settings für CI-Patienten angepasst.

### Findungsphase

Das zugrunde liegende Problem in der Findungsphase war, den individuellen Entwicklungsstand von Patienten in reproduzierbare und messbare Leistungen zu übersetzen: Für eine KI-gestützte App müssen die einzelnen Teilbereiche des Hörens also durch berechenbare Items messbar gemacht werden. Das Hören als Sinnesfunktion besitzt auf natürlich gegebene Art und Weise Kompetenzen, die sich im Verlauf der Hörrehabilitation idealerweise (wieder) weiterentwickeln und ausgebaut werden. Diese Kompetenzen dienen als Grundlage für die Entwicklung des vorliegenden Kompetenzmodells. In der Findungsphase wurden für den Bereich Hören im klinischen Setting der CI-Nachsorge für Erwachsene geeignete Kompetenzen ausgewählt. Die Auswahl der Kompetenzen stellt einzelne Fähigkeiten der Patienten als Leistungen heraus. Diese können später in der Performanz gemessen werden. Die Auswahl geeigneter Kompetenzen erfolgte in Experteninterviews mit dem therapeutischen Fachpersonal. Dabei wurde v. a. eine Gewichtung der einzelnen Teilbereiche des Hörens ermittelt und die mögliche Umwandlung von inhärent liegenden Kompetenzen der Patienten in messbare Teilleistungen im Rahmen der Implementierung in den KI-Service diskutiert. Aus dem Bereich Hörverstehen wurden die Kompetenzen Geräuschverstehen, das Laut‑, Wort- und Satzverstehen sowie die Orientierung im Raum ausgewählt. Weitere wichtige Faktoren für die Auswahl waren Aspekte der Umsetzbarkeit aus technologischer und datenschutzrechtlicher Sicht.

### Entwicklungsphase

#### Kompetenzmodell.

Im Kompetenzmodell wurden die Fähigkeiten („Teilkompetenzen“) nach erfolgter Zerlegung der Gesamtkompetenz „Hören“ bzw. „Sprachverstehen“ in einer Baumstruktur dargestellt. Abhängigkeiten, hierarchische Strukturen und Gewichtungen wurden basierend auf den im analogen Therapiesetting etablierten Ebenen des Geräusch‑, Phonem‑, Wort- und Satzverstehens visualisiert. Die erbrachten Leistungen des Nutzers im Spielverlauf spiegeln die – durch das System angenommene – tagesaktuelle Kompetenz des Nutzers in den verschiedenen Teilbereichen wider. Das Kompetenzmodell diente als Grundlage für die Auswahl der Schwierigkeitsstufe der Übungsaufgaben im Aufgabenmodell.

#### Aufgabenmodell.

**Abb. 1:**
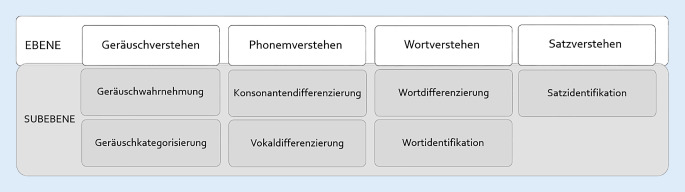
Ebenen des Aufgabenmodells mit Übungstypen

Um eine Teilkompetenz als Leistung messbar zu machen, bedarf es Aufgaben, die die Kompetenz des Patienten als messbare Leistung sichtbar machen [12]. Grundlegend für das Aufgabenmodell war die Berechnung der kumulativen Itemschwierigkeit, durch welche der Schwierigkeitsgrad einer Aufgabe bemessen werden konnte. Die Aufgaben, die durch den Übungsgenerator konstruiert wurden, bestanden aus je einem Zielitem und 3 Partneritems, die als Auswahlmöglichkeiten für den Patienten im User Interface (Definition s. Tab. 2) erschienen. Im Aufgabenmodell wurden entsprechend der vorab festgelegten Subkompetenzen jeweils bestimmte Aufgabentypen definiert (Abb. 1). Die Generierung der Übungsaufgaben erfolgte anhand festgelegter Parameter (Tab. 1) übungstypenspezifisch. Einheitlich wurden 5 Schwierigkeitsstufen festgelegt, in welchen die Partneritems mit dem Zielitem durch Wenn-dann-Bedingungen verknüpft wurden.

**Tab. 1:** Übersicht über die Assets und deren linguistische Eigenschafen

Vokale		Konsonanten		Wörter		Sätze	
Eigenschaft		Eigenschaft		Eigenschaft		Eigenschaft	
*Vokalklasse*	Frontal	*Konsonantenklasse*	Plosiv	*Phonemanzahl*	[Zahl]	*Wortanzahl*	[Zahl]
	Zentral		Frikativ/Affrikate				
	Hinten		Nasal, Lateral, Approximanten				
	Diphthong						
*Vokallänge*	Lang	*Konsonantenlänge*	Lang	*Silbenanzahl*	[Zahl]	*Satztyp*	Deklarativsatz
	Kurz		Kurz				Interrogativsatz
							Imperativsatz
*Sonorität*	Niedrig	*Stimmhaftigkeit*	Stimmhaft	*Vorkommenshäufigkeit*	Sehr häufig	*Satzstruktur*	Einfach
	Mittel		Stimmlos		Häufig		Mittel
	Hoch				Mäßig		Komplex
					Selten	*Verbvalenz*	Transitiv
					Sehr selten		Ditransitiv
							Intransitiv
						*Tempus*	Präsens
							Vergangenheit
							Futur
						*Modus*	Indikativ
							Konditional
						*Genus verbi*	Aktiv
							Passiv

#### Evidenzmodell.

Das Evidenzmodell bildete die Brücke zwischen der Performanz des Nutzers und dem Kompetenzmodell. Es beschreibt mathematisch, wie die Aktionen des Nutzers innerhalb einer Aufgabe (Wahl der korrekten oder nichtkorrekten Antwort) auf die einzelnen Kompetenzen einzahlen. Die Annahmen der Teilkompetenzen im individuellen Kompetenzmodell des Patienten passten sich für die Auswahl des Schwierigkeitsgrads der nächsten Aufgabe entsprechend an.

#### Assetpool.

**Abb. 2:**
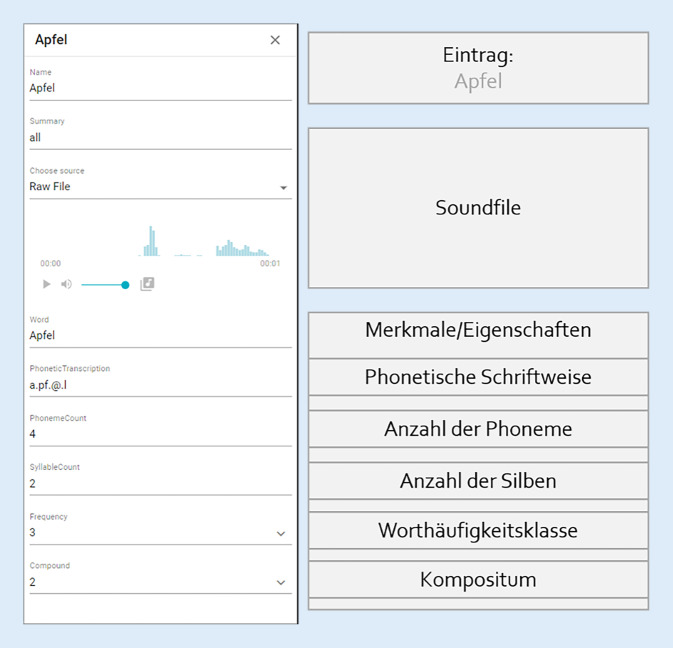
Beispiel eines Eintrags im Assetpool auf Wortebene

Der Übungsgenerator benötigte zur Erstellung einer Aufgabe neben einer Information zur Auswahl des Übungstyps auch die Information, welches Zielitem jeweils mit welchen Partneritems zusammengestellt werden musste, um den durch den KI-Service geforderten Schwierigkeitswert zu liefern. Aus diesem Grund wurden Assets (Definition s. Tab. 2) erstellt, in welchen die Items im sog. Assetpool mit Merkmalen und Eigenschaften beschrieben wurden, aus denen sich der Schwierigkeitsgrad des einzelnen Items berechnen ließ (Abb. 2). Aus der Kombination der Merkmale des einzelnen Items und der Zusammenstellung der Items in der Übungsaufgabe berechnete sich der Schwierigkeitswert der Übungsaufgabe.

**Tab. 2:** Definition der genutzten technologischen Begriffe

Assets	Ursprünglich: Vermögenswerte; hier: Aufstellung der Bewertung der Sound- und Sprachitems
Client	Vom Nutzer genutzte Softwarekomponente, die die Schnittstelle zwischen Datenaufbereitung und Datenerfassung bietet
Dashboard	Benutzeroberfläche zur Ansicht der Patientendaten in übersichtlicher Aufteilung
DiGA	Digitale Gesundheitsanwendungen sind digitale Medizinprodukte niedriger Risikoklassen, die die Versicherten etwa bei der Behandlung von Erkrankungen oder dem Ausgleich von Beeinträchtigungen unterstützen können [4]
User Interface	Benutzeroberfläche, auf der der Patient das Hörtrainingssystem bedient
Serious Game	Applikation mit spielerischem Charakter, die primär einen Bildungszweck hat

## Ergebnisse

**Abb. 3:**
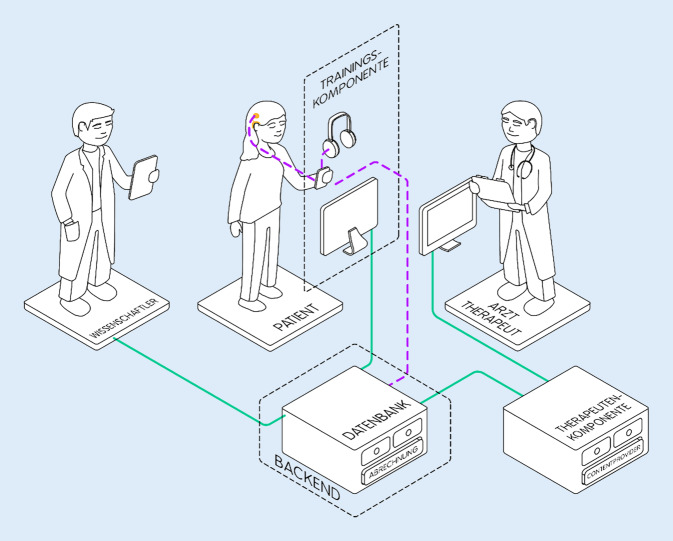
Komponenten des Trainingssystems im Projekt ProWear:Cochlea

Nach der Konzeption erfolgte die Umsetzung der einzelnen Komponenten der App in der Umsetzungsphase. Neben dem adaptiven Übungsgenerator und dem KI-Service wurden aufgrund der beschriebenen Anforderungen weitere Komponenten eingebunden. Dies umfasste die Entwicklung eines Spielumfelds inklusive Einstufungstest sowie des Dashboards für das Patientendaten-Management (Abb. 3).

### Dashboard.

**Abb. 4:**
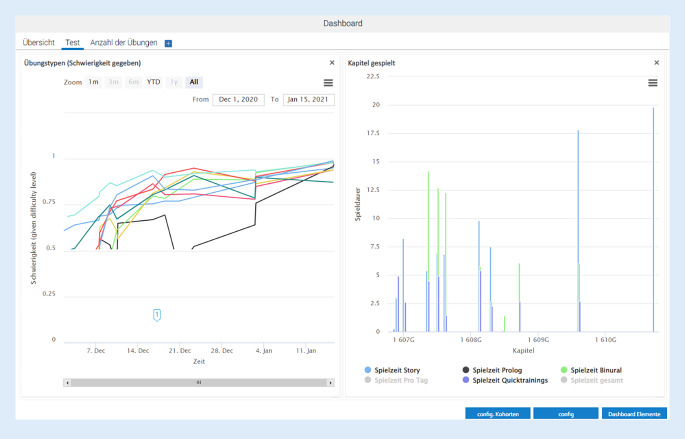
Ausschnitt aus dem Dashboard zur Supervision des Spielverlaufs eines Patienten (*links *Angabe des gegebenen Schwierigkeitsgrads der Aufgabe (gemittelt) für den Spielzeitraum (Datum), *rechts *Anteil der gespielten Teilbereiche der App, Angabe in Spieldauer/Datum)

Das Dashboard (Definition s. Tab. 2) umfasste verschiedene Ebenen: eine Übersichtsseite über die Nutzer, eine Ebene zum Ausfüllen der demografischen und anamnestischen Daten und eine Übersichtsseite, auf der die Ergebnisse aus den gespielten Übungen in Form von Diagrammen visualisiert wurden (Abb. 4). Im Dashboard wurden Daten zur Nutzungsdauer (Spielzeit), zur Anzahl der gespielten Übungen und der korrekten Antworten sowie des ausgegebenen Schwierigkeitswerts der Aufgaben visualisiert. Das Dashboard wurde unter Berücksichtigung hörtherapeutischer Ziele und datenschutzrechtlicher Vorgaben entwickelt und umgesetzt.

### Einstufungstest.

**Abb. 5:**
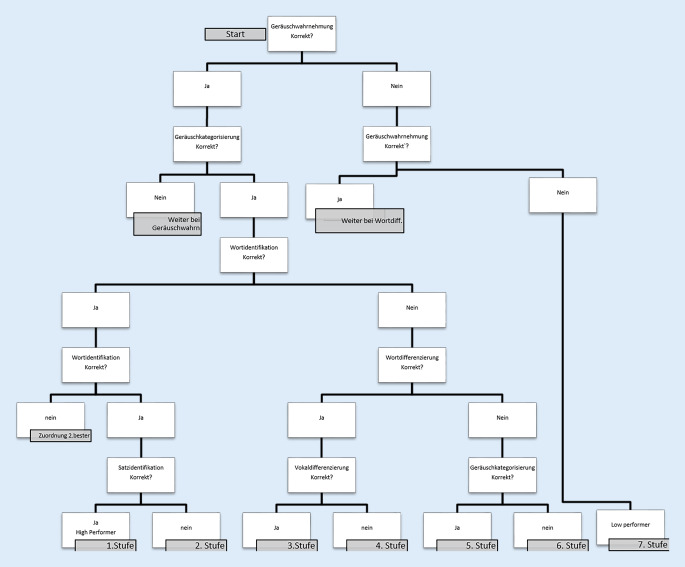
Algorithmus des Einstufungstests

In die Trainingskomponente wurde ein Einstufungstest integriert. Dieser diente der Messung der initialen Hörkompetenz und somit der Ermittlung eines Startwerts, der durch den KI-Service im ersten Übungsblock ausgegeben wird. Der Einstufungstest wurde im Sinne einer möglichst patientenorientierten und zeiteffizienten Durchführung so gestaltet, dass er den Patienten je nach Leistungsvermögen unterschiedliche Aufgaben zur Abfrage ihres Hörvermögens präsentierte. Wurde eine Aufgabe erfolgreich absolviert, so wurde die nächst schwierigere Aufgabe im Anschluss angeboten. Eine falsch beantwortete Aufgabe bedeutete im Anschluss die Präsentation einer einfacheren Aufgabe. Die Testaufgaben entsprachen den Übungstypen aus dem Aufgabenmodell (Geräuschwahrnehmung und -differenzierung, Lautidentifikation, Wortdifferenzierung und -identifikation und Satzidentifikation). Für die Einstufung wurden 7 Leistungsklassen von „Low Performer“ bis zu „High Performer“ definiert (Abb. 5).

### Client und User Interface.

**Abb. 6:**
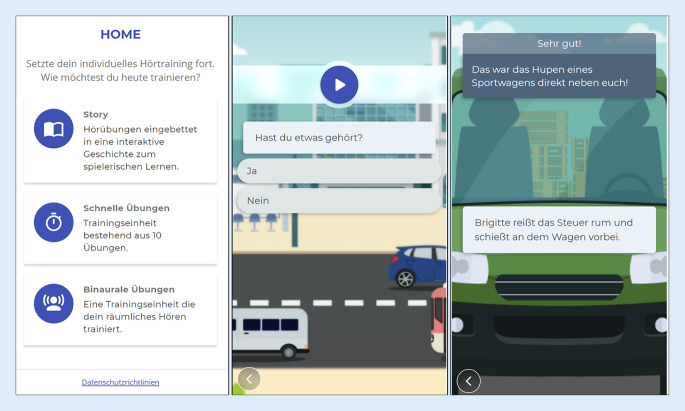
Start-Screen und Story-Telling-Format mit Beispiel einer Höraufgabe und Auflösung

Eine Benutzeroberfläche (User Interface) wurde webbasiert für Android und iOS-Systeme entwickelt (Definition s. Tab. 2). Nach Absolvieren des Einstufungstests hatten die Patienten die Wahl zwischen einem weiblichen und einem männlichen Avatar in Form eines CI-Trägers. Im Anschluss erschien ein Screen zur Auswahl des Übungsmodus: In den „Quicktrainings“ wurden 10 Höraufgaben im Block hintereinander präsentiert, im „Story-Telling-Format“ konnten die Höraufgaben in einer Geschichte über 10 Kapitel hinweg gespielt werden. Ein weiteres Modul mit Höraufgaben für binaural mit CI versorgte Patienten wurde außerdem angeboten (Abb. 6).

### Einbindung des adaptiven Systems.

Die Übungen in den Quicktrainings wurden durch den KI-Service jeweils für 10 Aufgaben im Block generiert und an das aktuelle Leistungsniveau des Nutzers angepasst. Im Story-Telling-Format wurden neben den Höraufgaben (Abb. 6) auch Entscheidungsfragen präsentiert und durch den Patienten beantwortet, anhand derer die Geschichte unterschiedliche inhaltliche Wendungen erhielt. Die Entscheidungsfragen wurden nicht vom Leistungsniveau beeinflusst, sondern waren fest im Story-Telling-Format integriert. Mithilfe des Avatars wurde der Nutzende auf diese Weise zum aktiven Teilnehmenden der Geschichte.

### Sound- und Sprachmaterial.

Insgesamt wurden 1053 Sound- und Sprachdateien (Items) im Assetpool eingetragen. Der Assetpool wurde durch eine Eingabemaske bedient, die die Merkmale und Eigenschaften der Sound- und Sprachdateien abfragte. Diese wurden durch geschultes, wissenschaftliches Personal mit entsprechenden linguistischen Kenntnissen editiert (Tab. 1). Die Sounddateien setzten sich aus lizenzfreien Downloads entsprechender Datenbanken zusammen [10, 11]. Die Auswahl umfasste Geräusche verschiedener Kategorien wie z. B. Tiergeräusche, Musikinstrumente, Natur oder Umwelt. Die Sprachdateien umfassten Vokale und Konsonanten, Wörter sowie Sätze, die durch eine geschulte Sprecherin im schallarmen Raum mit professioneller Hard- und Software eingesprochen und anschließend durch einen Toningenieur kontrolliert und in der Lautstärke reguliert wurden.

Die Berechnung der Schwierigkeit der Sound- und Sprachdateien erfolgte mithilfe zuvor bestimmter Merkmale, die in der Linguistik zumeist in experimentellen Settings genutzt werden [18, 22]. Wie bereits mehrfach in anderen Kontexten beschrieben, z. B. in audiometrischen Hörtestungen, erfolgte eine Eingabe deskriptiver Merkmale, wie die Einstufung von Vokalen und Konsonanten in Klassen oder das Zuordnen von grammatischen Markern auf Wort- und Satzebene. Zudem wurde auf Lautebene die akustische Dauer (Länge), bei Vokalen die Sonorität und bei Konsonanten die Stimmhaftigkeit bewertet. Auf Wortebene wurde die Phonem- und Silbenanzahl gezählt und die Worthäufigkeit (Vorkommenshäufigkeit) laut Duden in einer 5‑stufigen Einteilung angegeben [8]. Auf Satzebene wurde, neben der Anzahl der Wörter pro Satz, auch der Satztyp und die Satzstruktur (eingeteilt in die 3 Stufen einfach – mittel – komplex) beurteilt. Zudem wurden weitere Kriterien wie die Verbvalenz, Tempus, Modus und Genus verbi auf Satzebene bestimmt (Tab. 1).

### Klinische Studie

Eine klinische Machbarkeitsstudie wurde durch die Ethikkommission der Heinrich-Heine-Universität Düsseldorf positiv bewertet (Studien-Nummer 2020-880) und gemäß den ethischen Grundsätzen der „Deklaration von Helsinki“ am Hörzentrum der HNO-Klinik des Universitätsklinikums Düsseldorf durchgeführt. Ein Eintrag in das Deutsche Register Klinischer Studien (DRKS) erfolgte unter der Nummer DRKS00022860. Der Prototyp wurde von CI-Patienten aus dem Hörzentrum Düsseldorf über 4 Wochen im häuslichen Gebrauch getestet (*n* = 26). Die erfolgreiche Umsetzung des technologischen Konzepts, bestehend aus den beschriebenen Komponenten und den entsprechenden Schnittstellen, zeigte sich im störungsfreien Betrieb über den gesamten Testzeitraum. Erste Auswertungen der Ergebnisse geben Hinweise darauf, dass Patienten v. a. ein effizientes und zielgerichtetes digitales Hörtraining wünschen und ein gutes Handling im Gebrauch des Trainingssystems zeigen. Eine hohe Erfolgsquote in den Aufgaben sowie eine insgesamt lange Nutzungsdauer geben erste Hinweise auf eine hohe Akzeptanz des vorgestellten Prototyps.

## Diskussion

Das ECD wird bereits in schulischen Kontexten erfolgreich angewendet [7]. Im klinischen Kontext der Hörrehabilitation konnte das ECD erstmals als Rahmen für die Entwicklung eines Hörtrainingssystems angewendet werden. Das ECD zielt dabei darauf ab, ein Testdesign zu entwickeln, das valide Evidenz für die Einschätzung der Kompetenzen der zu testenden Personen liefert. Je komplexer der Prozess ist, diese Fähigkeiten zu bemessen, desto hilfreicher ist ein Rahmen, der Kompetenzen, Leistungen und Evidenz miteinander in Verbindung setzt [9, 19, 20]. Im deutschsprachigen Raum sind adaptive Lernsysteme im Bereich der Hörrehabilitation noch nicht etabliert [26]. Somit findet sich in diesem Projekt erstmals die Verknüpfung eines adaptiven technologischen Designs mit einem Trainingssystem, das sich auf das Wiedererlernen der inhärenten menschlichen Kompetenz „Hören“ bezieht. Die Vorzüge des Konzepts zeigten sich in der Beurteilung der Hörkompetenz sowie in der präzisen Einschätzung der dynamischen Leistungen. Zudem ermöglicht es eine Transparenz für die an der Entwicklung beteiligten Experten, sodass eine Interpretation der Ergebnisse erleichtert wird, wie auch Shute et al. beschreiben [21]. Die Anpassung des Designs an die klinischen Anforderungen von Fachpersonal und Patienten konnten auf verschiedenen Ebenen miteinander vereinbart werden. Das hier vorgestellte System umfasst die von Henshaw et al. beschriebenen Vorteile eines computerbasierten Hörtrainings [13]:

- nichtortsgebundenes Training,
- auf die individuellen Bedürfnisse der Patienten zugeschnittene Trainingseinheiten,
- Remote-Monitoring über das Dashboard,
- Datenerfassung über das Internet, Datenspeicherung auf gesicherten Servern.

Der Einsatz personalisierter digitalisierter Trainingsprogramme ist im Bereich der Serious Games ein erprobtes Verfahren, um langfristige Erfolge in der Behandlung verschiedener Störungsbilder zu erzielen (z. B. M. Parkinson, Depressionen) [14]. Serious Games können auf Entwicklungen aus dem Gaming-Bereich zurückgreifen und so z. B. mittels personalisierter Strategien auf die Zielgruppe zugeschnitten und entsprechend gezielt evaluiert werden [5]. Die hier vorgestellten, auf Basis des ECD entwickelten Komponenten wie das Kompetenz- und das Aufgabenmodell schaffen insbesondere im Bereich Hörstörungen verschiedene Anwendungsmöglichkeiten. Im Gegensatz zu herkömmlichen, statischen Hörtrainingsprogrammen bieten adaptive Systeme Anpassungsmöglichkeiten in der Anwendung. Dabei können sowohl der Schwierigkeitsgrad linear oder dynamisch angepasst werden als auch individuelle Merkmale wie z. B. Begleiterkrankungen in den KI-Service integriert werden. Diese Chancen sind nur in der Entwicklung adaptiver Verfahren zu finden [12, 25].

Die erforderliche detaillierte Auseinandersetzung mit dem Sound- und Sprachmaterial auf der Ebene des Aufgabenmodells wurde bisher in der Literatur nicht beschrieben. Die systematische Erfassung der Itemmerkmale über eine Maske und die Berechnung der kumulativen Itemschwierigkeit wurde bislang in audiologisch-sprachtherapeutischen Settings nicht genutzt. Die Berücksichtigung der Worthäufigkeit ist ebenfalls in audiometrischen Verfahren und linguistischen Experimenten gängig, der Einsatz im Rahmen sprachtherapeutischer Interventionen ist dagegen selten beschrieben. Die hier genutzte Worthäufigkeit auf Basis der Angaben im Duden bietet eine anwendungsfreundliche Zuordnung in 5 Häufigkeitsklassen und ist frei zugänglich [8]. Der Dudenkorpus umfasst derzeit mehr als 6 Mrd. Wortformen aus verschiedensten Quellen. In experimentellen Settings häufig genutzte Korpora wie CELEX2-DE oder SUBTLEX-DE geben eine absolute Vorkommenshäufigkeit der jeweiligen Wörter an, sie verarbeiten zumeist jedoch nur ausgewählte Quellen wie Literatur oder Untertitel und stellen somit eine deutlich geringere Abbildung des gegenwärtigen Sprachgebrauchs dar [1, 2]. Ein weiterer bisher wenig beachteter Faktor in der Berechnung der Itemschwierigkeit in digitalen Trainingsprogrammen ist die phonologische Nachbarschaftsgröße. Dieser wurde im vorgestellten Projekt erstmalig als ein wichtiger Indikator für die Unterscheidung der Level auf Wortebene mit einbezogen [23].

## Ausblick

Die Nachsorge von CI-Patienten kann durch ein adaptives digitales Hörtrainingssystem in einem kontinuierlichen, interaktiven Prozess unter Berücksichtigung individueller Bedürfnisse gewinnbringend erweitert werden. Das anhand des ECD-Frameworks entwickelte vorgestellte Design kann als Vorlage für benutzerbasierte, anpassungsfähige, digitale Hörtrainingsprogramme auch in internationalen Kontexten angewendet werden. Die Methode der Berechnung der Itemschwierigkeit bedarf sprachspezifischer Anpassungen, die durch linguistisch-sprachtherapeutisches Fachpersonal durchgeführt werden können. Grundlagenforschung im Bereich der Festlegung linguistischer Parameter ist dafür essenziell.

## Fazit für die Praxis

- Die Nutzung vorhandener Modelle zur Einbindung intelligenter, adaptiver Systeme ist im klinischen Kontext möglich und lässt sich aus anderen Kontexten heraus abbilden.
- Die Kontrolle und Berechnung der Items mithilfe linguistischer Merkmale ist notwendig, um entsprechende Algorithmen formulieren zu können.
- Ein zeiteffektives und patientenorientiertes Training ist nicht nur zeitgemäß, sondern auch aus ökonomischen und personalschonenden Aspekten zukunftsweisend.
- Weitere evidenzbasierte Grundlagenforschung muss erfolgen, um adaptive Verfahren anwenderorientiert gestalten zu können.
