# Erratum zu: Konzept und Umsetzung eines adaptiven digitalen Hörtrainingssystems für die Cochlea-Implantatnachsorge

**DOI:** 10.1007/s00106-024-01531-x

**Published:** 2024-11-07

**Authors:** Maika Werminghaus, Florian Gnadlinger, Jutta G. Richter, André Selmanagić, Susann Thyson, Dorothee Schatton, Thomas Klenzner

**Affiliations:** 1 Klinik für Hals-Nasen-Ohrenheilkunde, Hörzentrum, Medizinische Fakultät und Universitätsklinikum Düsseldorf, Heinrich-Heine-Universität Düsseldorf, Moorenstr. 5, 40225 Düsseldorf, Deutschland; 2 Masrayk University, Brünn, Tschechien; 3 Klinik für Rheumatologie, Medizinische Fakultät und Universitätsklinikum Düsseldorf, Heinrich-Heine-Universität Düsseldorf, Düsseldorf, Deutschland; 4 Hiller Forschungszentrum Rheumatologie, Medizinische Fakultät und Universitätsklinikum Düsseldorf, Heinrich-Heine-Universität Düsseldorf, Düsseldorf, Deutschland; 5 Forschungs- & Weiterbildungszentrum für Kultur und Informatik, Hochschule für Technik und Wirtschaft Berlin, Berlin, Deutschland

**Erratum zu:**

**HNO 2023**

10.1007/s00106-023-01414-7

In diesem Artikel hätte im Abschnitt „Ergebnisse/Klinische Studie“ in dem Satz beginnend mit „Ein Eintrag in das Deutsche Register Klinischer Studien (DRKS) …“ die Nummer „2020025397“ richtigerweise „DRKS00022860“ lauten sollen.

Der Originalbeitrag wurde korrigiert.

